# Effects of prey, pitcher age, and microbes on acid phosphatase activity in fluid from pitchers of *Sarracenia purpurea* (Sarraceniaceae)

**DOI:** 10.1371/journal.pone.0181252

**Published:** 2017-07-18

**Authors:** Carl S. Luciano, Sandra J. Newell

**Affiliations:** Department of Biology, Indiana University of Pennsylvania, Indiana, PA, United States of America; Pennsylvania State University, UNITED STATES

## Abstract

Carnivory in pitcher plants generally involves digestion of prey, by the plant itself, by symbionts, or both. While symbionts appear to be important in the digestion of prey in *Sarracenia purpurea*, the importance of pitcher-derived enzymes is less well documented. Our goal was to reduce microbial numbers in pitcher fluid in order to measure the acid phosphatase activity attributable to the pitchers themselves. Preliminary experiments indicated that various antibiotics were minimally effective at reducing microbial populations and that antibiotic-resistant microbes were easily cultured from pitcher fluid. Consequently, we measured the abundance of culturable microbes in every sample taken for the measurement of acid phosphatase activity. Pitchers fed with one sterilized ant had higher levels of acid phosphatase activity than unfed pitchers. Older pitchers were more responsive to feeding than young pitchers. Pitchers with high levels of microbes (on Day 5) had higher acid phosphatase activity than pitchers with low levels of microbes. However, fed pitchers were not more likely to have higher microbe levels and microbe levels were not related to pitcher age. When fluid samples from inside the pitcher were compared to appropriate controls incubated outside the pitcher, acid phosphatase activity was higher inside the pitcher. Results from the feeding experiments are consistent with a primary role of microbes in the digestion of prey in pitchers of *S*. *purpurea*. However, the relationship between pitcher age and enzyme activity is not a function of microbes in the pitcher fluid and may depend on enzymes produced by the plant. Our methods would not detect microbes embedded on the inner surface of the pitcher; and if they survived the alcohol rinse and antibiotics, we cannot rule out microbes as the source of the relationship between pitcher age and acid phosphatase activity.

## Introduction

Carnivory evolved independently in several plant families resulting in a diversity of trapping mechanisms such as the snap trap of *Dionaea muscipula*, the suction trap of *Utricularia*, the sticky-leaf trap of *Drosera* and *Pinguicula*, and the pitfall trap of the various genera of pitcher plants [1–4]. In all types of traps, carnivory generally involves digestion of prey. Digestion may be accomplished by the plant, by its symbionts, or both [5,6]. Some studies of digestion in pitcher plants have recognized the importance of distinguishing between enzymes produced by plants and enzymes produced by microbial symbionts.

There are pitcher plants that clearly produce digestive enzymes in the pitchers. *Nepenthes* species are well studied in this regard. For example, Jentsch [7] purified the protease nepenthacin, and Tökés et al. [8] reported proteases and lipase in *Nepenthes* secretions taken from sterile closed pitchers. More recently, Rotloff et al. [9] isolated a class III acid endochitinase from closed pitchers of *Nepenthes* species. In the most extensive study to date, Buch et al. [10] measured protease activity in several *Nepenthes* species and the plant's response to prey introduction. However, even in *Nepenthes* digestion may be facilitated by bacterial symbionts [11].

Other pitcher plants are less well studied, but there is evidence for plant-produced digestive enzymes in other genera. Using enzyme labeled fluorescence (ELF), Płachno et al. [12] demonstrated the activity of phosphatases on the walls of gland cells on the inner surface of pitchers of *Cephalotus follicularis* and *Nepenthes tobaica*. However, they also noted phosphatase activity associated with bacteria on the inner wall of *Cephalotus* pitchers and in the pitcher fluid of both *Cephalotus* and *Nepenthes*. Closed pitchers of *Heliamphora tatei* contained fluid that exhibited protein hydrolytic activity, but four other species of *Heliamphora* tested negative [13]. *Heliamphora* and *Sarracenia* are closely related [14], and *Darlingtonia californica*, which is sister to the *Sarracenia-Heliamphora* clade, apparently has no digestive glands and makes no digestive enzymes [15]. The possible presence of plant-produced digestive enzymes in *Sarracenia* is interesting from an evolutionary perspective since some related species appear to differ in their digestive processes.

In *Sarracenia* much early work assayed for digestive activity in pitcher fluid of open pitchers [16], although some studies distinguished between fluid taken from open or closed pitchers [17]. Since unopened pitchers are sterile [18,19], this should effectively rule out a microbial source of digestion. After pitchers open they are eventually colonized by a diverse assemblage of bacteria [19]; and, depending on food web structure, bacteria increase in density and richness when nutrients are added (*e*.*g*., [20–24]). Using a cytochemical stain, Stauffer [25] demonstrated acid phosphatase activity in sessile glands on both the outside and inside surface of *S*. *purpurea* pitchers as well as in the pitcher fluid, but he did not rule out the potential for bacterial enzymes. *Sarracenia purpurea* pitchers also had measurable activities of protease, RNase, nuclease, and phosphatase when sampled a week after adding antibiotics [26]. However, we suggest caution in interpreting the source of this enzyme activity since bacteria may repopulate the pitcher after the addition of antibiotics. Recently, Fukushima et al. [27] assayed pitcher fluid from four species and found digestive proteins; but the potential for microbial sources was not investigated.

The extent to which *S*. *purpurea* pitchers produce digestive enzymes is still an open question [28]. Acid phosphatase activity in pitcher fluid may be important for several reasons. Phosphorus is one of several limiting nutrients for *S*. *purpurea*, and insect prey provide phosphorus to the plant [29–32]. Pitcher fluid is often slightly acidic [28], and Stauffer [25] found glands on the surface of *S*. *purpurea* leaves that stained positive for acid phosphatase activity. Extracellular acid phosphatase hydrolyzes phosphate from phosphoesters, making phosphorus from organic sources more readily available to the plant; and extracellular acid phosphatase may derive from plants or microbes [33–35].

Since *S*. *purpurea* pitchers are open to rainfall, a diverse aquatic community forms within the confines of a pitcher. Inquiline communities of *S*. *purpurea* pitchers may include bacteria, archaea, algae, fungi, protists, rotifers, cladocerans, copepods, mites, mosquito larvae (*Wyeomyia smithii*), midge larvae (*Metriocnemus knabi*), and sarcophagid fly larvae (*Fletcheromyia fletcheri*); and the composition and relative abundances of these organisms is highly variable among pitchers and among geographical locations [19,36–38]. Much research has focused on the dipterans. For example, Heard [39] described the relationship between dipteran larvae, *W*. *smithii* and *M*. *knabi*, and *S*. *purpurea* as mutualistic, demonstrating that the *M*. *knabi* larvae initiate the breakdown of prey by physically tearing the carcass, making more nutrients available to other inquilines and to the pitcher.

However, microbes appear to play a pivotal role in the digestive capacity of *S*. *purpurea*. *Sarracenia purpurea* pitchers with and without complete inquiline communities did not differ in their nitrogen uptake [40]. And, Butler et al. [40] demonstrated that nitrogen uptake by *S*. *purpurea* pitchers was similar to nitrogen uptake by pitchers of *Sarracenia alata* and *Sarracenia flava*. Unlike *S*. *purpurea*, neither *S*. *alata* nor *S*. *flava* supports a complex aquatic community, but like *S*. *purpurea* both are exposed to bacterial colonization. Koopman et al. [41] and Koopman and Carstens [42] found a rich bacterial community within *S*. *alata* pitchers, one that was quite different from the microbial community of the surrounding soil yet mirroring the population genetic structure of the plant species. Mouquet et al. [43] modeled the inquiline ecosystem within *S*. *purpurea* pitchers and concluded that bacteria alone would maximize the availability of nitrogen to the plant and that additional components of the food web would likely influence the plant via their effect on the bacteria. Clearly, the bacterial community is important for digestion of prey in *Sarracenia* pitchers.

Does *S*. *purpurea* rely on symbiotic organisms such as bacteria for the production of digestive enzymes? What is the plant’s capacity for digestion of prey? Initially our goal was to quantify the amount of enzyme activity deriving from the plant itself by preventing the colonization of new pitchers by bacteria or eliminating bacteria from older pitchers. We experimented with several antibiotics to determine their effectiveness. After it became clear that antibiotics were not necessarily effective in eliminating microbes in the fluid of the pitcher, we used an ethyl alcohol rinse and antibiotics to try to enhance the inhibition of microbes in the pitcher. Our initial goal of eliminating the bacteria was unrealistic, as we document below. Our modified goal was to sufficiently reduce the bacterial numbers for a short period of time to be able to quantify the enzyme activity of the pitcher.

More specifically, we ask the following questions:

1. How best can we eliminate or reduce the impact of microbes on enzyme activity so that measurements reflect the enzyme activity of the pitchers themselves?
2. Does feeding the pitcher increase the enzyme activity within the pitcher?
3. Is enzyme activity related to pitcher age? Are older pitchers more responsive to feeding than younger pitchers?
4. Within the context of the experiment (where we have attempted to reduce microbes), are microbe levels correlated with either initial or final enzyme activity levels? Do samples with higher microbe levels have higher levels of enzyme activity? Do older pitchers or fed pitchers have higher levels of microbes?
5. Can we distinguish the relative importance of microbes vs. pitchers for the acid phosphatase activity? Do outside controls exhibit enzyme activity? In other words, when samples are removed from the pitcher on Day 0 and incubated for 5 d outside the pitcher, do they exhibit the same patterns of enzyme activity as samples incubated inside the pitchers?

## Methods

*Sarracenia purpurea* L. [44] were obtained from a commercial nursery as adult plants and maintained in a growth chamber under controlled conditions for several years prior to the experiments. Growth medium was an unsterilized soil mixture of 6 parts commercial peat, 2 parts perlite, and 1 part sand. Day:night schedule in the chamber was 14 h:10 h photoperiod and 23 C:15 C temperature regime. Relative humidity was at or above 60%. Plants were routinely monitored for pitcher formation and pitcher age, and pitchers were used in experiments as they became available. Margins of immature pitchers are sealed and thus the interiors are microbiologically sterile until the margins separate and the pitchers open [19]. To prevent aerial colonization by microorganisms following opening, individual pitchers were covered with sterile plastic Whirl-Pak bags prior to opening. Bags were suspended over pitchers by attaching the bag to two slender stakes, forming a tent over the entire pitcher. The bag was placed over the pitcher shortly before it opened and remained on the pitcher for the duration of the experiment. Raising the bag on the stakes subsequently allowed access to the pitcher during experiments. The bag was not sealed around the base of the pitcher, and no condensation occurred in the bags.

Ants were used as simulated prey animals. Ants for prey were purchased from Carolina Biological Supply Company (“black wood ants, species not identified”) and stored at -20 C until used. Ant carcasses were sterilized by autoclaving for 15 min at 121 C.

All chemicals, substrates, buffers and antibiotics were purchased from Sigma-Aldrich. Microbiological media included Nutrient Agar (NA), Corn Meal Agar (CMA), Luria-Bertani Agar (LB), Potato Dextrose Agar (PDA), and Trypticase Soy Agar (TSA). All microbiological media were purchased from Becton and Dickinson Company (BD) under a variety of brand names and prepared according to the supplier’s instructions.

The acid phosphatase assay was standard, following the directions provided with the substrate from Sigma-Aldrich. Acid phosphatase activity was assayed in 0.01 M MES (2[N-morpholino)ethanesulfonic acid), at pH 6.0 with 0.01 M 4-nitrophenylphosphate (Paranitrophenylphosphate or PNPP) as substrate. Assays were incubated at room temperature for at least 1.5 h. The reaction was then stopped by the addition of 1.0M NaOH. In qualitative assays results were assessed visually by the presence of yellow color in the sample, but in quantitative assays A405 nm was measured in a 96-well microplate format using a microplate reader with readings in μg/mL accurate to three decimal places. Assays were done in duplicate. Each sample was assayed twice in adjacent wells of a microtiter plate, and the two acid phosphatase measurements were averaged to provide one measurement per sample. Wheat germ acid phosphatase was used as a standard. Pitcher fluid was assayed for a small number of pitchers as the pitchers became available; fresh standards were used for every set of assays. Beckman Coulter Phi Series pH meters and combination electrodes were used for pH measurements.

### Preliminary experiments

In order to attribute enzyme activity to the pitcher, we needed to eliminate the possibility of enzyme activity by bacteria. Hence, we needed to establish the best methods for removal of microbes from pitchers and best possible conditions for microbial growth in order to be confident bacteria were not present in the samples. Initially, we tested various agars and incubation temperatures to determine the optimal conditions for growth of microbes derived from pitchers. Microbial cultures from two older pitchers, freely exposed to the air in the growth chamber for an extended time, were plated onto nutrient agar (NA) at pH 6.8, potato dextrose agar (PDA) at pH 5.8, Luria broth (LB) agar at pH 7.0, corn meal agar (CMA) at pH 6.0 and trypticase soy agar (TSA) at pH 7.3. These pHs result with standard preparation of these agars. Plates were incubated at 15 C (the low temperature setting of the growth chamber), 25 C (similar to the high temperature setting of the growth chamber), or 35 C (near the standard of 37 C for incubating bacteria) for four days. Numbers of colony forming units per plate (CFU/plate) were counted daily and averaged for each medium at each temperature.

Microbes were abundant in pitchers. In order to ascribe enzyme activity to the pitcher, we began a series of experiments to determine the effectiveness of antibiotics at reducing microbial populations. At the start of the experiments (n = 13 pitchers), endogenous pitcher fluid was removed and each pitcher received 8 mL of sterile water containing 250 μg/mL cefotaxime, 100 μg/mL carbenicillin, and 100 μg/mL ampicillin (CCA cocktail). Two mL samples were removed from each pitcher, starting on Day 0 and every other day through Day 8. Samples were assayed for acid phosphatase as described above. Also, for each sample pH was measured, and each sample was plated on NA and LB and incubated at 25 C for 4 d to check for microbial growth. Some pitchers received additional CCA cocktail on Days 2, 4, and 6. For these pitchers, all pitcher fluid was removed, and 2.5 mL of fresh antibiotic solution were placed in the pitcher. pH was measured and a sample removed (prior to replacing the antibiotics) for the acid phosphatase assay. Replacement of antibiotics did not produce different results, so data from this series of experiments have been combined.

When these antibiotics proved relatively ineffective, bacteria derived from 12 pitchers were plated in duplicate samples (i.e., from one loop) onto two additional plates of NA, one with and one without CCA cocktail to test for antibiotic resistance. *Escherichia coli* served as a control.

A second series of preliminary experiments (n = 31 pitchers) tested a different set of antibiotics. At the start of each experiment, each pitcher received PSA cocktail, a mixture of 10,000 units per mL of penicillin, 10 μg/mL of streptomycin and 25 μg/mL of the antimycotic amphotericin B (Sigma antibiotic/antimycotic lyophilized powder, A7292). The buffer was 10 mM solution of MES, calibrated to pH 6.0. This buffer has a useful pH range of 5.5–6.7 with a pKa of 6.1. The PSA cocktail (5–6 mL) was added to each pitcher within a couple days after the pitcher opened, as soon as the width of the pitcher opening allowed access. To determine whether bacteria were present in a pitcher, each sample was plated onto nutrient agar (NA) and incubated at 25 C for 3–4 d. Four of the 31 pitchers were also fed sterilized prey. To feed a pitcher one ant was included with the buffered antibiotics. Some pitchers were also subjected to repeated antibiotics after the start of the experiment. Experiments in this second series had similar results, so data from these preliminary experiments were combined.

Bacteria were isolated from 17 different pitchers and were tested for resistance to several antibiotics. Antibiotics included 1) a mixture of 20,000 units/mL penicillin + 20 μg/mL streptomycin + 50 μg/mL amphotericin B (which is 2x standard concentration), 2) 50 μg/mL tetracycline, 3) 150 μg/mL chloramphenicol, 4) 50 μg/mL gentamicin. Bacterial isolates were plated onto NA containing antibiotics and incubated at 25 C for 3 d. A control plate did not contain antibiotics.

The final set of preliminary experiments tested the combination of an ethanol rinse and PSA cocktail. The pitcher was filled with 70% (v/v) ethanol. The surface sterilant was removed after 5 minutes of exposure. The pitcher was filled with PSA antibiotic mixture in 10mM MES pH6. The pitcher was immediately plugged with sterile cotton to prevent microbial contamination. These experiments proceeded as in previous experiments, measuring acid phosphatase activity, pH, and microbial growth. This procedure produced minimum microbial growth and was used for the subsequent experiment. In the preliminary experiments results were assessed qualitatively; no statistical analyses were performed.

### Effect of prey, pitcher age, and microbes on acid phosphatase activity

In this experiment, endogenous pitcher fluid was removed from the pitcher and stored frozen. Based on preliminary experiments each pitcher was treated with ethanol followed by the PSA cocktail. Forty-six pitchers, ranging in age from 2–43 d since opening, were included in the unfed treatment. Fifty-six pitchers, ranging in age from 1–43 d since opening, were included in the fed treatment (i.e., fed prey of one sterile ant in sterile water). Pitchers were on different plants, generally similar in size, and large enough to contain the antibiotic cocktail. Pitchers were haphazardly assigned to the fed or unfed treatment, taking care to include a wide spread of pitcher ages in each group.

For each pitcher, acid phosphatase (AcP) activity was assayed several times, using the protocol described above for: 1) pitcher fluid sampled immediately after the fluid was placed in the pitcher on Day 0 (AcP(0)); 2) pitcher fluid sampled after 5 d inside the pitcher (AcP(5)); 3) fluid placed in the pitcher, immediately removed, and incubated in a test tube in the growth chamber for 5 d (AcP(o-5)); 4) fluid not placed in the pitcher, sampled at the start of the experiment (Initial buffer control); 5) fluid not placed in the pitcher, incubated in a test tube in the growth chamber for 5 d (Final buffer control). Fed pitchers had controls with and without an ant. Each sample was plated onto nutrient agar (NA) and incubated at 25 C for 3–4 d. Microbes(0) is the count of colony-forming units per plate (CFUs) in the Day 0 or initial sample. Microbes(5) is the count of CFUs in the Day 5 sample take from inside the pitcher. Microbes(o-5) is the count of CFUs in the Day 5 sample taken from the test tube outside the pitcher.

Simple statistical analyses, including descriptive statistics, regressions, and *t*-tests, were performed in Excel (Microsoft Office 2007, Microsoft Corporation, Redmond, WA). ANOVAs and nonparametric tests were performed in SPSS (IBM SPSS Statistics Version 23, Release 23.0.0.0, 2015, IBM Corporation, Armonk, NY).

## Results

### Preliminary experiments

Even with no opportunity to collect rainfall, growth chamber pitchers produced an endogenous fluid within the confines of the pitcher trap (Range = 0–2.2 mL). All samples of pitcher fluid contained culturable microorganisms though their number and colony morphology varied from pitcher to pitcher. Pitcher fluid microbes had diverse growth patterns in culture but we did not attempt to identify microbial species. In preliminary experiments we examined growth of pitcher fluid microbes on various standard growth media and at different incubation temperatures. Table 1 presents data from one pitcher in a representative experiment.

**Table 1:** Growth of microbes from endogenous pitcher fluid from one representative pitcher as measured in average number of colony-forming units per plate (average CFU/plate)^1^.

Incubation Temperature (C)	Medium^2^	Day 1	Day 2	Day 3	Day 4
35	CMA	0	2	20.5	23.5
	LB	1.5	11	29	36.5
	NA	30	69	TNTC^3^	TNTC
	PDA	0	12	22.4	28
	TSA	0	22.8	43	54
25	CMA	0	0	TNTC	TNTC
	LB	30.5	69	TNTC	TNTC
	NA	39	80.5	TNTC	TNTC
	PDA	9.2	41.6	TNTC	TNTC
	TSA	17.2	47.6	TNTC	TNTC
15	CMA	0	0	0.5	0.5
	LB	0	5	27	57.5
	NA	0	10.5	31	59.5
	PDA	0	0	13.2	28
	TSA	0	18.4	39	60

^1^ Duplicate 50 μL samples of endogenous pitcher fluid from growth chamber plants were plated on microbial media, incubated at indicated temperatures and scored for colony formation at daily intervals after inoculation. Data represent average values of the duplicate samples for one representative pitcher.

^2^ CMA: Corn Meal Agar, LB: Luria-Bertani Agar, NA: Nutrient Agar, PDA: Potato Dextrose Agar, TSA: Trypticase Soy Agar.

^3^ TNTC: too numerous to count

Microbes from endogenous pitcher fluid grew most rapidly at 25 C. On all five media tested at 25 C, colony numbers increased to a quantity too numerous to count (TNTC) by 72 h of incubation. In contrast, at 35 C only organisms plated on NA reached comparable levels, whereas at 15 C none of the samples on any medium tested reached levels too numerous to count even after 96 h of incubation.

Microbes from endogenous pitcher fluid had different growth rates on the five different media tested. At all three incubation temperatures tested, microbial growth was slower on CMA and PDA than on other media. At 25 C and 35 C growth was most rapid on NA, and at 15 C the growth rate on NA was approximately the same as the growth rate on TSA. Overall, NA at 25 C supported the most rapid rate of microbial growth. Based on this experiment, we selected nutrient agar (NA) and an incubation temperature of 25 C for additional experiments. LB agar was included for comparison with other studies using this medium [20,22,45].

In additional preliminary experiments we evaluated the effect of two antimicrobial cocktails on the growth of microorganisms in pitcher fluid. In some of these experiments, we removed endogenous fluid from the pitcher and replaced it with a CCA cocktail (as in [26]). Of thirteen samples removed from different pitchers 4 d after receiving CCA cocktail, 5 (38%) exhibited abundant microbial growth and 8 (62%) exhibited some microbial growth. In other experiments we replaced endogenous pitcher fluid with PSA cocktail prior to sampling and culturing. Of the fluid samples removed from 31 different pitchers 4 d after receiving PSA cocktail, 19 samples (61%) exhibited abundant microbial growth, 6 (19%) samples exhibited reduced microbial growth, and 6 (19%) samples exhibited no microbial growth. Neither cocktail (CCA or PSA) abolished the growth of microorganisms from inside the pitcher traps, but the PSA cocktail slowed microbial growth compared to the CCA cocktail.

Finally, in experiments with 28 pitchers we rinsed the inside of the pitcher trap with 70% ethanol prior to the addition of PSA cocktail and blocked the pitcher opening with a sterile cotton plug prior to sampling and culturing. In these experiments, 7 (25%) fluid samples showed abundant microbial growth, 8 (29%) showed reduced levels of growth, and the remaining 13 (46%) showed no microbial growth. The combination of ethanol rinse followed by PSA cocktail produced the greatest reduction in microbial growth.

When bacteria derived from 12 pitchers were plated in duplicate samples on NA with and without CCA cocktail, 11 samples exhibited microbial growth on NA+antibiotics. One pitcher sample and the *E*. *coli* control did not grow on the NA+antibiotics. In a mixture 2x the standard concentration of PSA in nutrient agar, 15 of 17 isolates grew. Fifteen isolates grew on nutrient agar infused with 50 μg/mL tetracycline. Fourteen isolates grew on nutrient agar infused with either 150 μg/mL chloramphenicol or 50 μg/mL gentamicin. All isolates grew on the control NA without antibiotics.

### Effects of prey, pitcher age, and microbes on acid phosphatase activity

**Fig 1:**
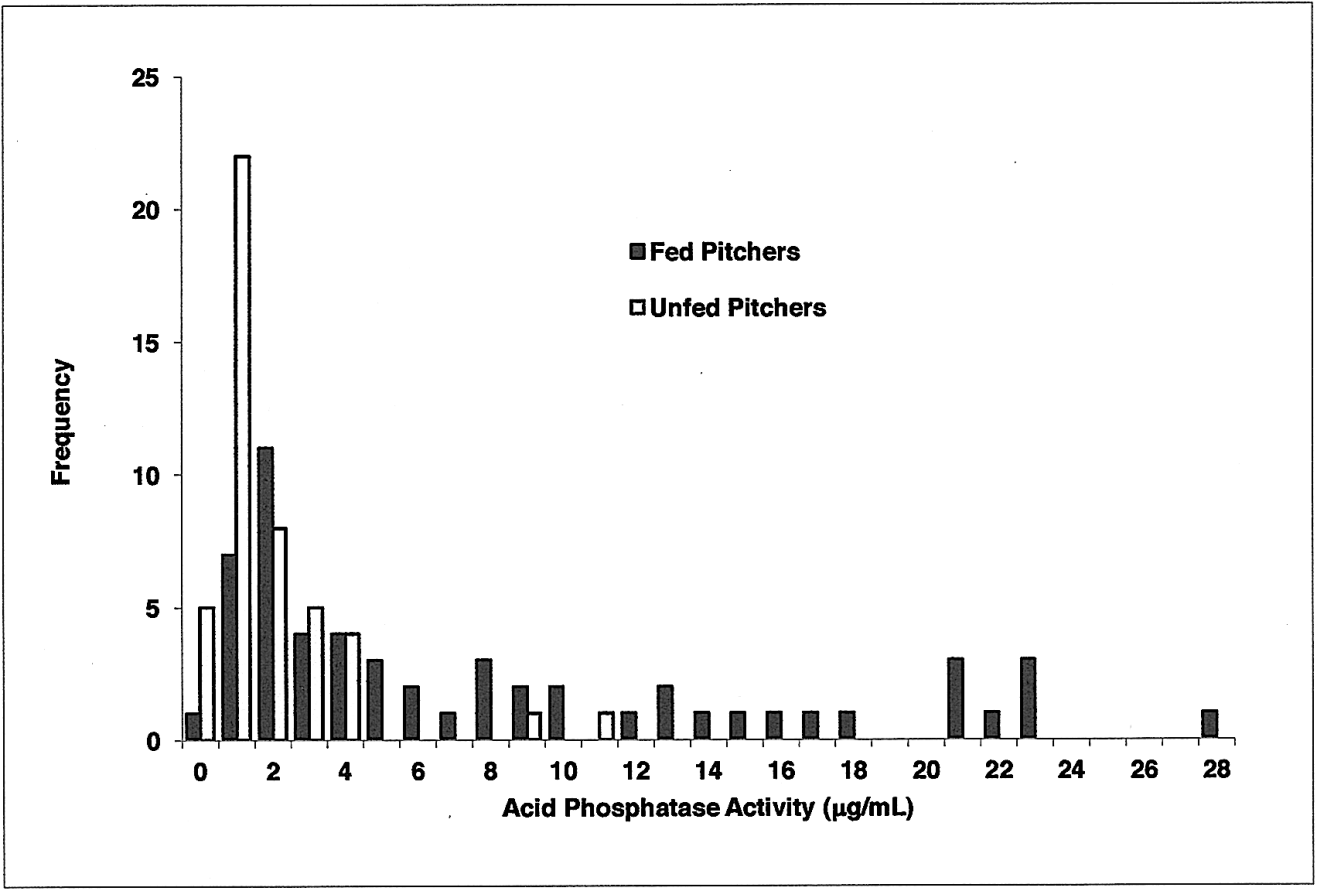
Frequency distribution illustrating the number of pitchers with a given level of acid phosphatase activity (μg/mL) inside the pitcher on Day 5 of the experiment (AcP(5)). Fed pitchers (black bars) exhibited a more skewed distribution with higher levels of acid phosphatase activity than unfed pitchers (gray bars).

Pitchers fed one sterilized ant on Day 0 generally had higher levels of acid phosphatase activity on Day 5 (AcP(5)) than unfed pitchers, and fed pitchers exhibited a skewed frequency distribution of acid phosphatase activity (Fig 1 and S1 Supplementary Information). Log transformation of the data allowed for a one-tailed *t*-test assuming unequal variances (P = 5.6 x 10^−10^) with antilog means: fed pitcher mean = 4.69 μg/mL (95% confidence interval = 3.38–6.39 μg/mL; n = 56) vs. unfed pitcher mean = 0.98 μg/mL (95% confidence interval = 0.66–1.36 μg/mL; n = 46). A Mann-Whitney U test also indicated that fed pitchers had significantly different distribution of data than unfed pitchers (P < 0.001).

Final acid phosphatase activity inside the pitchers, measured on Day 5 of the experiment (AcP(5)), was significantly higher than initial acid phosphatase activity, measured on Day 0 of the experiment (AcP(0)), for both fed and unfed pitchers. A paired t-test for the unfed pitchers, using log-transformed data, indicated that AcP(5) of 0.98 μg/mL is significantly greater than AcP(0) of 0.12 μg/mL (one-tailed P = 4.83 x 10^−10^). Similarly, in fed pitchers AcP(5) of 4.69 μg/mL is significantly greater than AcP(0) of 0.17 μg/mL (one-tailed P = 1.02 x 10^−18^).

**Fig 2:**
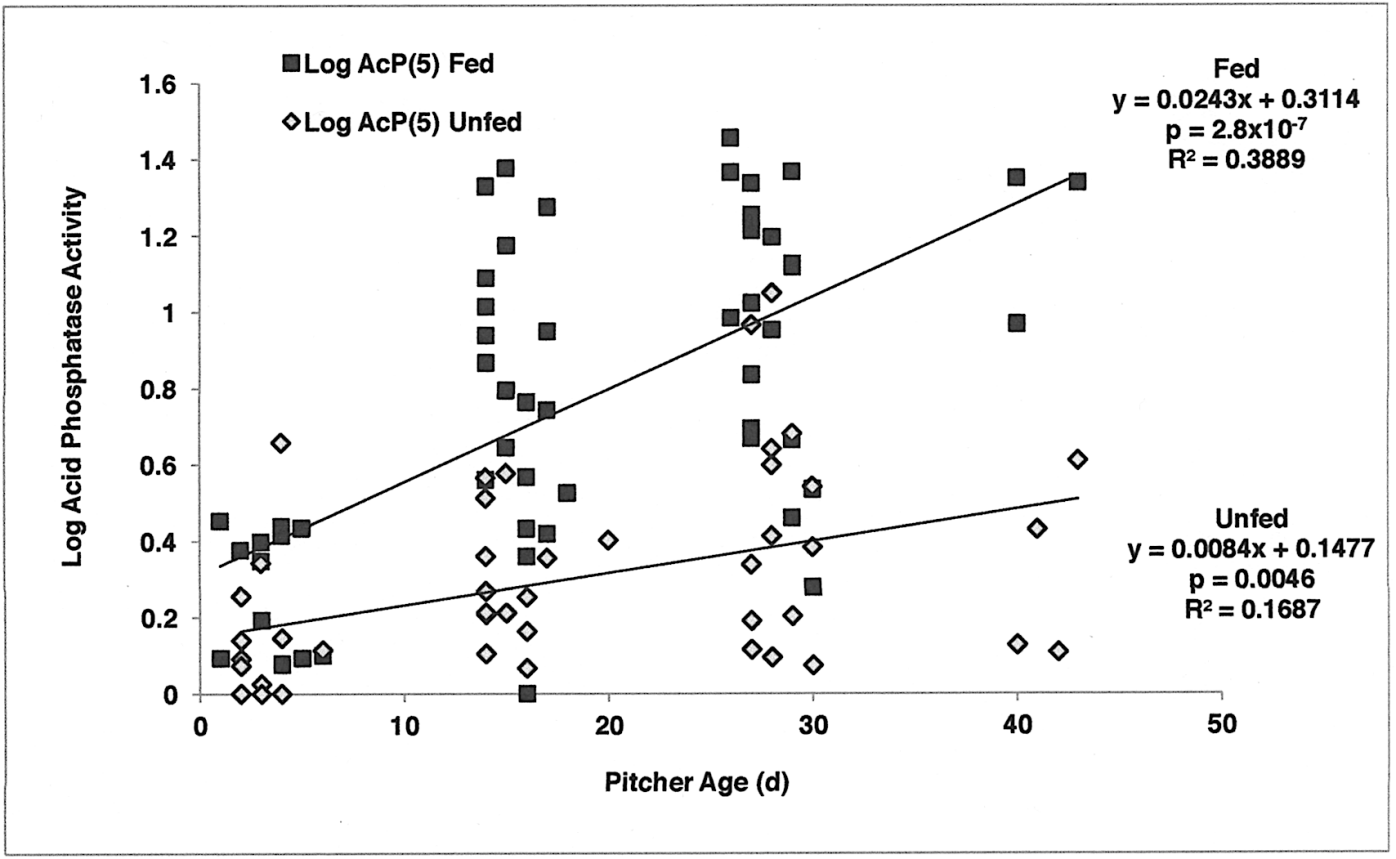
Regression analysis of log acid phosphatase activity across pitcher age (d) for fed and unfed pitchers. Both fed and unfed pitchers exhibited an increase in acid phosphatase activity with increasing pitcher age, with a greater increase in the fed pitchers. Pitcher age accounted for almost 39% of variation in enzyme activity in fed pitchers and just under 17% of the variation in enzyme activity in unfed pitchers.

Regression analysis indicated that initial acid phosphatase activity (Log AcP(0)) was very weakly related to pitcher age (R^2^ = 0.0763). Final acid phosphatase activity (Log AcP(5)) generally increased with pitcher age (Fig 2). Pitcher age accounted for almost 39% of the variation in acid phosphatase activity on Day 5 in fed pitchers but less than 17% of the variation in acid phosphatase activity in unfed pitchers (using log-transformed data). Pitchers older than 6 d more readily responded to feeding (Fig 2).

**Fig 3:**
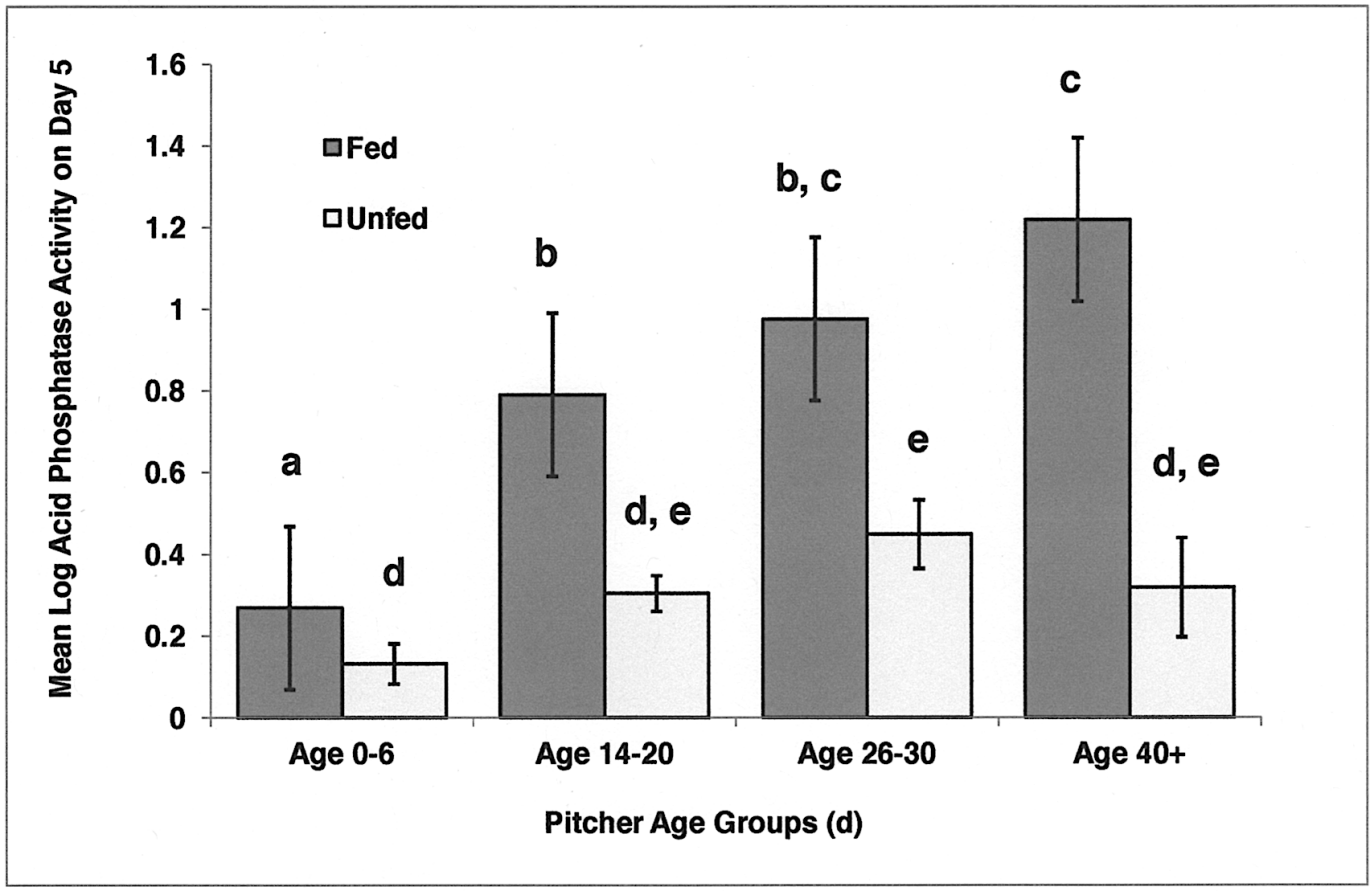
Means (± 1 SE) of the log of acid phosphatase activity measured on Day 5 (Log AcP(5)) for fed and unfed pitchers in four age groups. Fed pitchers exhibited a trend of increasing enzyme activity with pitcher age. Homogeneous subsets identified by Tukey HSD are labeled with the same letter. Sample sizes for Fed are: Age 0–6, n = 13, Age 14–20, n = 21, Age 26–30, n = 19, Age 40+, n = 3; and for Unfed are: Age 0–6, n = 14, Age 14–20, n = 14, Age 26–30, n = 14, Age 40+, n = 4.

As Fig 2 illustrates, pitcher ages fall into four categories: 0–6 d, 14–20 d, 26–30 d, and 40+ d. Log of final acid phosphatase activity (Log AcP(5)) data were sorted into these four age groups. In fed pitchers, a one-way ANOVA of Log AcP(5) demonstrated significant differences across four age groups (P < 0.001) (Fig 3). Acid phosphatase activity increased with increasing age of the pitcher (Fig 3). In unfed pitchers, a one-way ANOVA of Log AcP(5) demonstrated significant differences across four age groups (P = 0.008); however, Levine’s test indicated a lack of homogeneity of variances (P = 0.042). The youngest pitchers had relatively low levels of acid phosphatase activity, but there is not an overall trend with age (Fig 3). A 2-way ANOVA (Feeding Status + Age + Interaction) was precluded in that error variances were unequal based on Levine’s Test (P = 0.010).

**Fig 4:**
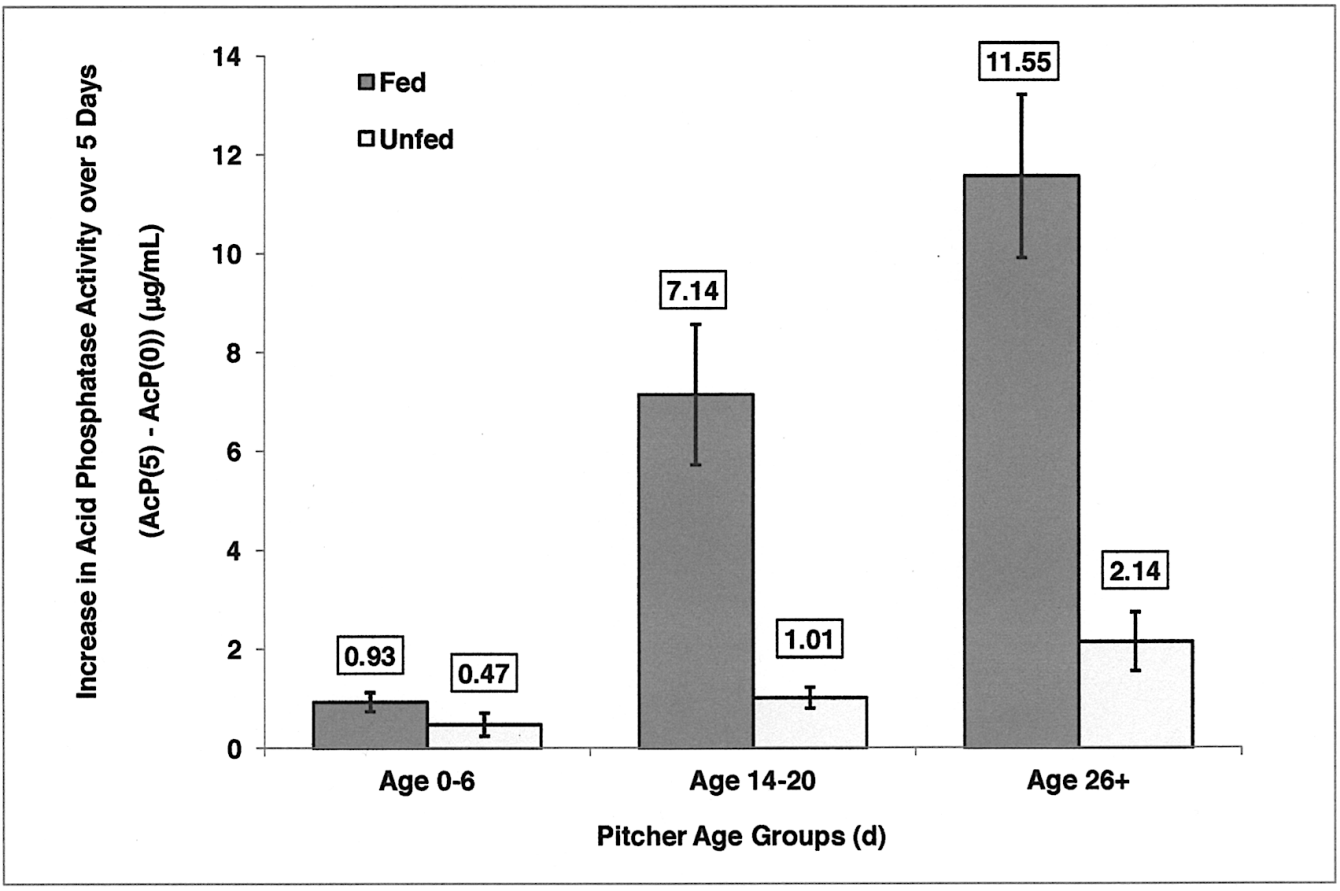
Initial acid phosphatase activity (AcP(0)) (μg/mL) subtracted from final acid phosphatase activity (AcP(5)) μg/mL) in fed (dark gray bars) and unfed (light gray bars) pitchers for three age groups of pitchers. Differences (mean ± 1 SE) increased with pitcher age, to a greater degree in fed pitchers than in unfed pitchers. (Samples sizes are: Age 0–6, Unfed, n = 14, Fed, n = 13; Age 14–20, Unfed, n = 14, Fed, n = 21; Age 26+, Unfed, n = 18, Fed, n = 22).

Are older pitchers more responsive to feeding than younger pitchers? For this analysis, we used the differences between final and initial measurements of acid phosphatase activity (AcP(5)-AcP(0)). Because of the small samples sizes of the oldest pitchers, they were combined into a group 26+ d old. The data, both raw and transformed, failed to meet the assumption of homogeneous variances required by a 2-way ANOVA. Therefore, AcP(5)-AcP(0) values were analyzed using the nonparametric test of Friedman’s two-way analysis of variance by ranks comparing across three age groups and across two groups of feeding status, and the null hypothesis was rejected (P = 0.003) Older pitchers responded more readily to feeding than younger pitchers (Fig 4).

**Fig 5:**
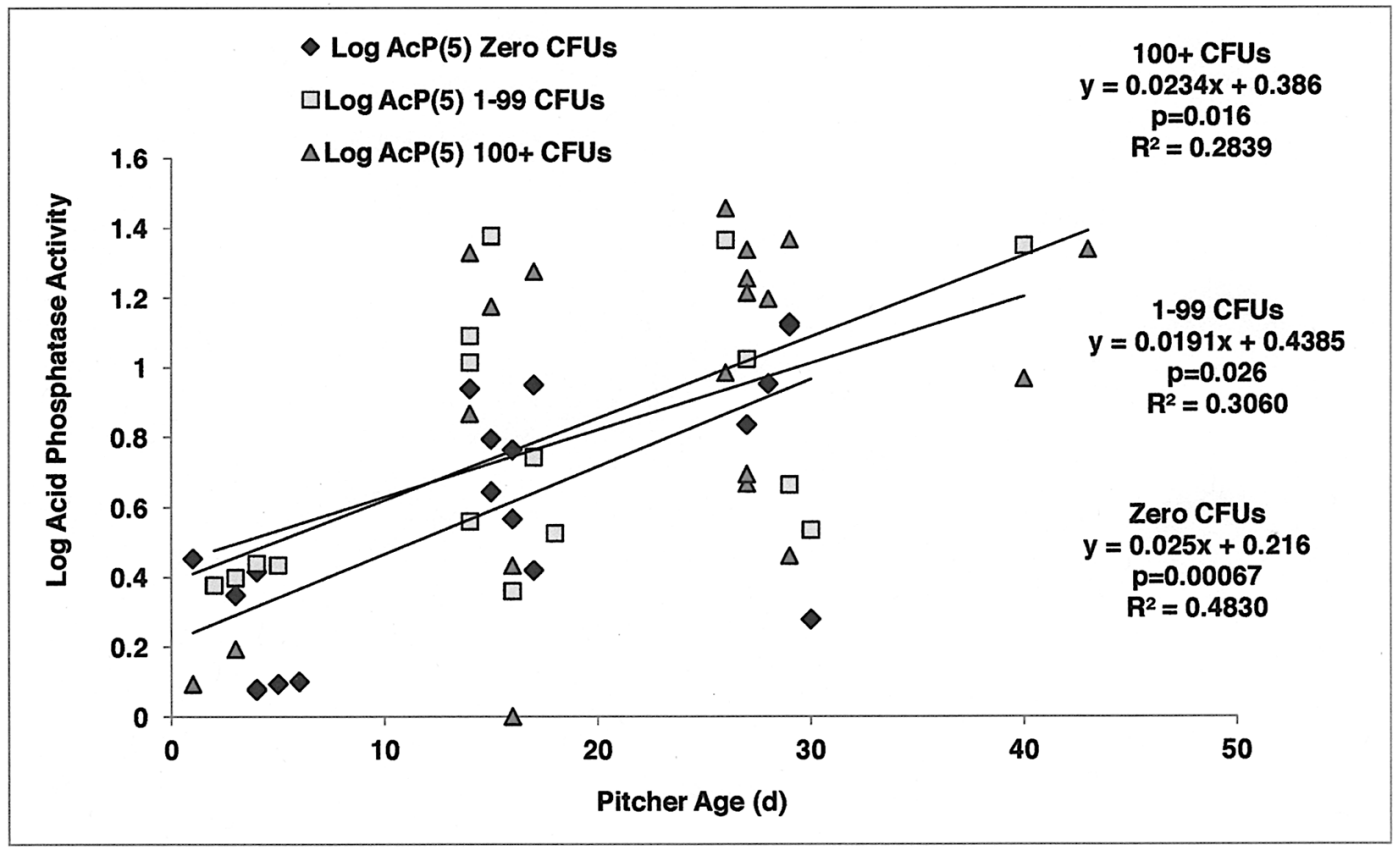
Regression analysis of log acid phosphatase activity on Day 5 across pitcher age (d) for fed pitchers separated on the basis of microbe levels on Day 5 (Microbes(5)), taken from samples of pitcher fluid. Enzyme activity increased with pitcher age in all three microbe levels. The amount of variation explained by pitcher age (R^2^) decreased with increasing microbe levels.

In spite of efforts to control microbial populations, 32% of pitchers produced culturable microbes on Day 0, and 56% of pitchers produced culturable microbes at Day 5. Sixty-four percent of fed pitchers produced culturable microbes, whereas 46% of unfed pitchers produced culturable microbes at Day 5. Microbe levels at Day 0 (Microbes(0) measured in CFUs) were very weakly related to pitcher age (R^2^ = 0.0605). Also, microbe levels at Day 5 (Microbes(5) measured in CFUs) were very weakly related to pitcher age (R^2^ = 0.0782). In regression analyses of samples grouped on the basis of Day 5 microbe levels (Microbes(5): Zero CFUs; 1–99 CFUs; 100+ CFUs), log acid phosphatase activity increased with pitcher age for all groups (Fig 5). Pitcher age explains more variation in AcP activity for samples with lower microbe levels. R^2^ values for Zero CFUs, 1–99 CFUs, and 100+ CFUs are 48%, 30%, and 28%, respectively.

Are microbe levels related to enzyme activity within the pitcher? Regression analysis indicated that initial acid phosphatase activity (Log AcP(0)) was not related to Microbes(0) (R^2^ = 0.0147). Final acid phosphatase activity (Log AcP(5)) was very weakly related to Microbes(5) in unfed pitchers (R^2^ = 0.0697) and in fed pitchers (R^2^ = 0.1265).

For statistical analyses samples were grouped into two categories: High Microbes(5) in which CFUs on Day 5 were equal to or greater than 100; and Low Microbes(5) in which CFUs on Day 5 were less than 100 (including zero CFUs). High Microbes(5) samples had significantly higher levels of AcP(5) than Low Microbes(5) samples (one-tailed *t*-test assuming unequal variances, on log-transformed data, P = 0.003; Mann Whitney U test P = 0.007). Antilog mean of acid phosphatase activity in High Microbes(5) samples = 4.46 μg/mL (95% confidence interval = 2.66–7.16 μg/mL, n = 31) and in Low Microbes(5) samples = 1.92 μg/mL (95% confidence interval = 1.39–2.58 μg/mL, n = 71).

Do fed pitchers have higher microbe levels? There were 20 samples that were Fed and had High Microbes(5), 11 samples were Unfed, High Microbes(5), 36 were Fed, Low Microbes(5), and 35 were Unfed, Low Microbes(5). A G-test of independence using Williams’s correction for small sample size results in Gadj = 1.652 (P > 0.05), indicating that microbe levels and feeding status were independent. Fed pitchers were not more likely to have higher microbe levels.

**Fig 6:**
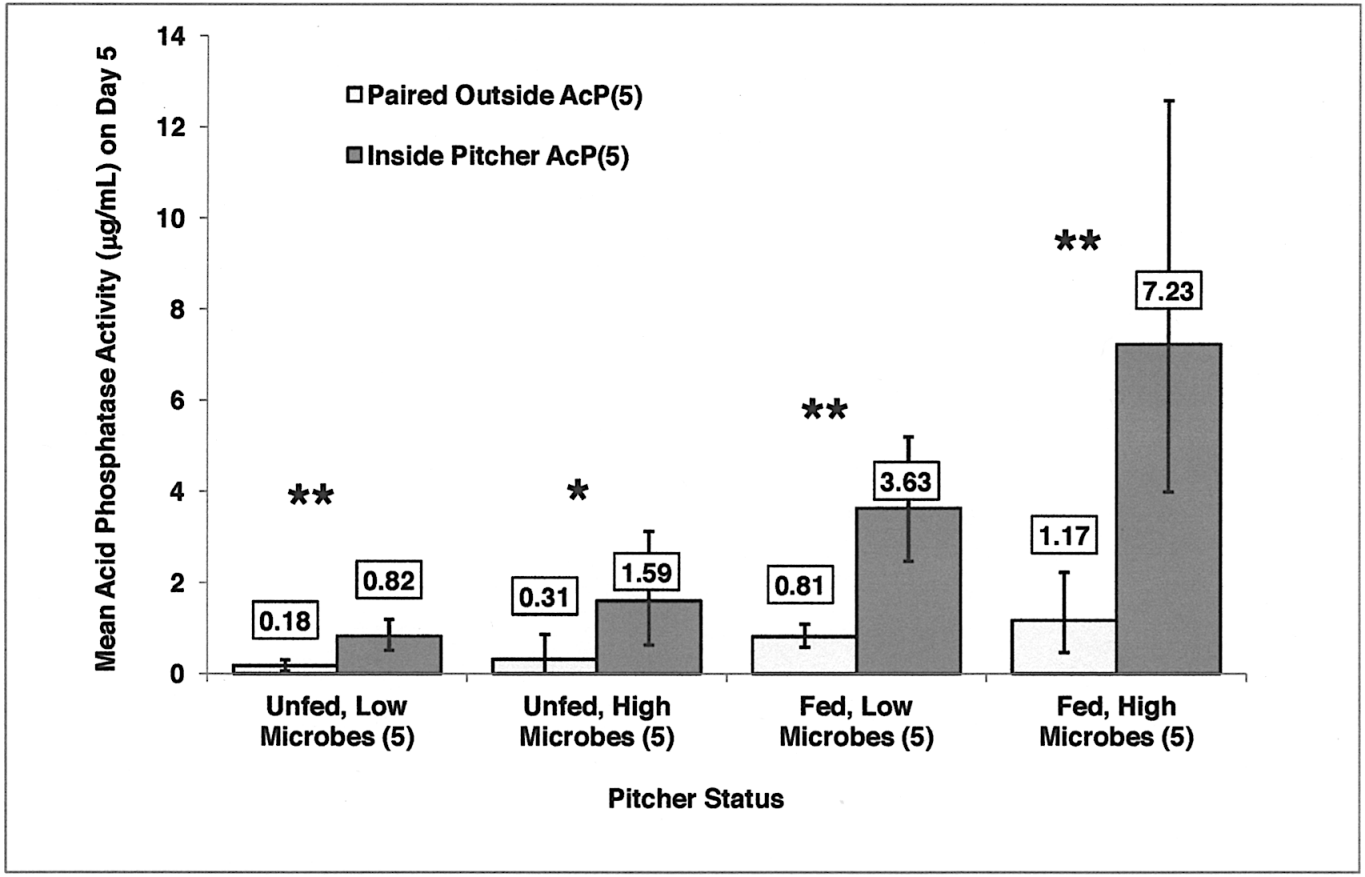
Mean acid phosphatase activity of fluid inside the pitcher, measured on Day 5 (Inside Pitcher AcP(5)), was significantly higher than paired controls. Samples taken from each pitcher on Day 0 were incubated outside the pitcher, and acid phosphatase activity was measured on Day 5 (Paired Outside AcP(5)). Paired Outside AcP(5) and Inside Pitcher AcP(5) are sorted by feeding status and microbe levels inside the pitcher on Day 5 (Microbes (5)). The asterisks represent a statistically significant difference between Paired Outside AcP(5) and Inside Pitcher AcP(5) using paired t-tests, * = P < 0.01, ** = P < 0.001. Paired t-tests were performed on log-transformed data, and antilogs of means are represented with 95% confidence intervals. (Sample sizes are: Unfed, Low Microbes (5), n = 35; Unfed, High Microbes (5), n = 11; Fed, Low Microbes (5), n = 36; Fed, High Microbes (5), n = 20).

When samples were removed from the pitcher on Day 0 and incubated for 5 d outside the pitcher, did they exhibit the same amount of enzyme activity as samples incubated inside the pitchers? To address this question, we did two separate analyses. In the first analysis, samples were grouped into categories based on feeding status and the quantity of microbes found inside the pitcher on Day 5 (Microbes(5), measured in CFUs). This resulted in four groups: Unfed, Low Microbes(5); Unfed, High Microbes(5); Fed, Low Microbes(5); Fed, High Microbes(5). Within each of the four groups, a paired t-test comparing the enzyme activity inside the pitcher (Inside AcP(5)) with paired samples incubated outside the pitcher (Paired Outside AcP(5)) resulted in a significant difference (Fig 6). Inside AcP(5) samples were higher in acid phosphatase activity than Paired Outside AcP(5) samples (Fig 6).

In the second analysis, samples were grouped into categories based on feeding status and the quantity of microbes found on Day 5 in the samples incubated outside the pitcher (Microbes(o-5)). There were four groups analogous to the ones in the previous analysis. Paired *t*-tests comparing the enzyme activity outside the pitcher (Outside AcP(5)) with paired samples incubated inside the pitcher (Paired Inside AcP(5)) indicated significant differences in two of the four groups. In the Fed, Low Microbes(o-5) group, the Paired Inside AcP(5) mean = 4.19 μg/mL (95% confidence interval = 2.93–5.86 μg/mL) was significantly greater than the Outside AcP(5) mean = 0.74 μg/mL (95% confidence interval = 0.56–0.95 μg/mL) (n = 49, P = 2.035 x 10^−10^). In the Unfed, Low Microbes(o-5) group, the Paired Inside AcP(5) mean = 0.92 μg/mL (95% confidence interval = 0.60–1.32 μg/mL) was significantly greater than the Outside AcP(5) mean = 0.16 μg/mL (95% confidence interval = 0.06–0.27 μg/mL) (n = 41, P = 1.077 x 10^−5^). Very few samples incubated outside the pitcher fell into the High Microbes (o-5) groups (Fed, High Microbes(o-5), n = 5; Unfed, High Microbes(o-5), n = 4). Mean enzyme activity was higher in the samples from inside the pitchers, but confidence intervals were extremely large. Lack of significant differences between inside and outside samples in these two groups is likely due to the small sample sizes.

## Discussion

The initial goal of the preliminary experiments was to determine how best to eliminate microbes from the inside of the pitchers so that we could measure the acid phosphatase produced by the pitcher itself. Our approach differed from previous studies in that every sample of pitcher fluid was not only assayed for enzyme activity but also plated onto agar for microbial counts. Of the five agars tested, nutrient agar (NA) resulted in the most numerous CFUs. Pitcher-derived bacteria grew best at 25 C, consistent with incubation temperatures used in previous experiments (e.g., [20]). This culturing technique does not definitively indicate the absence of microbes [19,46] however, it certainly can indicate the presence of microbes.

Two combinations of antibiotics were tried, and in each series of experiments the pitchers were inoculated either at the start of the experiment or repeatedly every other day. Using a combination of antibiotics including carbenicillin, cefotaxime, and ampicillin (CCA cocktail) at the start of the experiment, microbes were present by Day 2. With the same combination of antibiotics given repeatedly every other day, microbes were present by Day 4. In another experiment we used a combination of buffered penicillin, streptomycin and amphotericin B (PSA cocktail) at the start of the experiment. Again, microbes were present within days; and renewing the antibiotics every other day did not change the results. This is in marked contrast to findings in *Nepenthes*, in which microbial growth is inhibited by pitcher fluid [47]. However, the microbes used in the *Nepenthes* study were not microbes naturally associated with *Nepenthes*; and this study may not reflect the relationship between *Nepenthes* and its associated microbes.

A final preliminary experiment demonstrated multiple microbial strains that were resistant to various combinations of antibiotics. Our results are consistent with Tran [48], who performed antibiotic sensitivity tests on strains of mycobacteria isolated from pitcher plant fluid. Tran's [48] results were mixed, with certain strains resistant to certain antibiotics and susceptible to others. In our study antibiotics did not effectively control microbial populations in the pitchers for more than a day or two. Unopened pitchers were entirely covered with sterile Whirl-pacs; although they were not in contact with the pitcher and not air-tight, a Whirl-pac would prevent air movement across the pitcher. We conclude that the most likely source of the microbes was the potting medium or the adjacent pitchers on the same plant. *Sarracenia purpurea* pitchers produce sugary nectar on the outside of the pitcher [49], and bacterial communities in pitchers are often limited by carbon availability [22]. We speculate that microbes arrive on the inside of the pitcher by way of the outside surface of the pitcher. Further exploration of the function of external nectar glands is warranted.

Prey, pitcher age, and presence of microbes all influenced the acid phosphatase activity in fluid from *S*. *purpurea* pitchers. The addition of prey increased acid phosphatase activity over that of unfed pitchers by almost 5x. Both fed and unfed pitchers increased acid phosphatase activity over the 5 d incubation period. Fed pitchers increased enzyme activity over 27x in 5 d, while unfed pitchers increased enzyme activity approximately 8x over the same time period. These results are consistent with the literature suggesting that enzyme production is induced by prey [26].

Fed pitchers exhibited a trend of increasing acid phosphatase activity with increasing pitcher age. Young pitchers (Ages 0–6 d) were far less capable of responding to prey than older pitchers. The second age group (Ages 14–20 d) increased enzyme production (final minus initial AcP) approximately 7x the increase of the first age group (Ages 0–6 d). The third age group (Ages 26+) increased enzyme production approximately 11x the increase of the first age group. Unfed pitchers exhibited less variation in acid phosphatase activity with pitcher age. Again, the youngest unfed pitchers had the lowest levels of enzyme activity. These results suggest that using newly opened pitchers for enzyme assays, in an effort to avoid microbial symbionts, may bias the measurement of enzyme activity toward the lowest values.

In general, pitchers with high levels of microbes exhibited over 2x higher levels of acid phosphatase activity than pitchers with low levels of microbes. Microbe levels at Day 0 and Day 5 were not a function of pitcher age, and fed pitchers were not more likely to have high microbes. When samples were grouped into four categories (Unfed, Low Microbes; Unfed, High Microbes; Fed, Low Microbes; Fed, High Microbes), all measurements of acid phosphatase activity inside the pitcher were significantly higher than the controls incubated outside the pitcher. In other words, the milieu inside the pitcher was necessary for enzyme production. The Fed, Low Microbe group exhibited approximately 4x the enzyme activity of the Unfed, Low Microbe group. Similarly, the Fed, High Microbe group showed a little over 4x the enzyme activity of the Unfed, High Microbe groups. The High Microbe groups and Low Microbe groups responded similarly to feeding. Microbes are generally capable of up-regulating enzyme production [35], and microbes can likely account for the increased level of enzyme activity within the pitcher fluid. Unless pitchers and microbes respond in the same way, these data suggest that microbes are responsible for the increase in acid phosphatase activity with the addition of prey.

Can we disentangle the relative importance of plant-derived enzymes from microbe-derived enzymes? The difficulty in eliminating microbes indicates that they are closely associated with the plant. They may be embedded in the waxy surface such that an ethanol rinse does not affect them. That microbes are responsible for some of the enzyme activity is apparent when the Low Microbe and High Microbe groups are compared. The fact that both Low Microbe and High Microbe groups responded similarly to feeding also suggests that the enzyme production being induced is that of microbes. However, fed plants were not more likely to have high levels of microbes, so feeding did not increase the population of microbes during the span of the experiment. This result contrasts with other experiments done in a natural setting, in which addition of prey resulted in higher levels of bacterial cells over a span of 15 d, a much longer time span than we used [21].

Our data on microbes do not explain all the patterns. In particular, the increase in enzyme activity with pitcher age appears to be unrelated to the level of microbes in the fluid of the pitchers. Microbe levels on Day 0 and Day 5 are not closely related to pitcher age. Also, in fed pitchers the trend of increasing enzyme activity with increasing pitcher age holds across all microbe levels. Older pitchers appear to be more competent to respond to prey. Young pitchers with high levels of microbes in pitcher fluid did not respond to feeding as strongly as older pitchers with lower levels of microbes.

However, our method does not rule out the presence of microbes that are undetectable by culturing [50] or that are embedded in the inner surface of the pitcher. Krieger and Kourtev [46] identified three distinct sub-habitats within pitchers of *S*. *purpurea*: the pitcher fluid, the bottom sediment, and the pitcher wall. They found high diversity of microbes within the pitcher using molecular techniques. In addition, the microbial populations differed among the three sub-habitats. Our pitchers had no bottom sediment since they were grown in a growth chamber. If microbes are sufficiently embedded in the waxy surface of the pitcher such that they unaffected by an ethanol rinse, our methods would not detect them. The pitcher wall and/or pitcher fluid may be repopulated after antibiotic treatments by microbes that are not culturable.

We hypothesize that older pitchers might have a more deeply embedded and more diverse community of microbes. Hence, the increase in acid phosphatase activity with pitcher age might be a function of the microbial community embedded in the pitcher wall, rather than a function of the microbes present in the pitcher fluid or of the pitcher itself. This would involve the process of succession in the bacterial community. Gray [51] described succession in the bacterial community within *S*. *purpurea* pitchers, and found a decrease in abundance and relatively constant species richness of culturable bacteria over several months. Using molecular techniques, Peeples and Kourtev [52] found diverse bacterial communities 48 h after the opening of pitchers. Over a two month period, bacterial communities in ten pitchers became more different. Further studies of enzyme activity on the pitcher wall, such as those by Stauffer [25] and Płachno et al. [12], could differentiate between enzyme production by the pitcher itself vs. by embedded microbes in pitchers of different ages.

Our data support the conclusion that microbes are important components of the digestion of prey by *S*. *purpurea* pitchers. The relative importance of the pitcher itself, beyond the maintenance of a habitat for symbionts, remains elusive.

## Supporting information

S1 Supplementary Information Complete data set of the main experiment. (XLSX)
